# Activity of T-type calcium channels is independent of CRMP2 in sensory neurons

**DOI:** 10.1080/19336950.2019.1608129

**Published:** 2019-04-26

**Authors:** Song Cai, Zhiming Shan, Zhongjun Zhang, Aubin Moutal, Rajesh Khanna

**Affiliations:** a Department of Pharmacology, College of Medicine, The University of Arizona Health Sciences, Tucson, AZ, USA; b Department of Anesthesiology, Shenzhen People‘s Hospital & Second Clinical Medical College of Jinan University, Shenzhen, P.R. China; c The Center for Innovation in Brain Sciences, The University of Arizona Health Sciences, Tucson, AZ, USA

**Keywords:** CRMP2, T-type calcium channel, DRG sensory neuron, electrophysiology

## Abstract

Amongst the regulators of voltage-gated ion channels is the collapsin response mediator protein 2 (CRMP2). CRMP2 regulation of the activity and trafficking of NaV1.7 voltage-gated sodium channels as well as the N-type (CaV2.2) voltage-gated calcium channel (VGCC) has been reported. On the other hand, CRMP2 does not appear to regulate L- (CaV1.x), P/Q- (CaV2.1), and R- (CaV2.3) type high VGCCs. Whether CRMP2 regulates low VGCCs remains an open question. Here, we asked if CRMP2 could regulate the low voltage-gated (T-type/CaV3.x) channels in sensory neurons. Reducing CRMP2 protein levels with short interfering RNAs yielded no change in macroscopic currents carried by T-type channels. No change in biophysical properties of the T-type currents was noted. Future studies pursuing CRMP2 druggability in neuropathic pain will benefit from the findings that CRMP2 regulates only the N-type (CaV2.2) calcium channels.

Regulators of voltage-gated calcium channels (VGCCs) shape a diversity of biological functions within the nervous system 1,2. In chronic pain, proteins regulating the N-type (CaV2.2) VGCCs can modulate nociception [1]. One such protein is the collapsin response mediator protein 2 (CRMP2) [3–15]. Our continuing studies have established CRMP2 as a bona fide binding partner and regulator of the presynaptic trafficking of CaV2.2 [4,8,13,15–18]. In neuropathic pain, increased phosphorylation of CRMP2 by cyclin-dependent kinase 5 defines the presynaptic content for CaV2.2, but not for CaV2.3 (i.e. the R-type) VGCCs [3,13,19]. In a proteomic study, the L-type VGCC was shown to be part of the CRMP2 interactome [20] likely though a motif in the C-terminus in the CaV1.2 L-type channel [21]. While CRMP2-dependent regulation of CaV2.1 (i.e. the P/Q-type) VGCC has never been investigated, CRMP2 interaction domains found in CaV2.2 are poorly conserved in CaV2.1 or other channels [21]. In investigating CRMP2 phosphorylation with the small molecule (*S*)-lacosamide, we found that only calcium influx via N-type, but not other high-voltage gated channels, was blocked [5]. Collectively, these results hinted at a *specific* action of CRMP2 on N-type VGCCs.

Loss of CRMP2 expression abolishes pathological pain [11,19]. This reversal of pain was attributed to decreased excitability of dorsal root ganglion (DRG) neurons likely due to CRMP2-dependent effects on the trafficking and activity of the NaV1.7 voltage-gated sodium channel [22]. Further work from our group revealed that a peptide derived from the CRMP2/CaV2.2 interface (designated CBD3, for calcium binding domain 3) decreased the frequency of spontaneous excitatory postsynaptic potentials in spinal cord [18]. A single mutation that stabilized CBD3’s helical structure rendered the mutant peptide capable of inhibition of sensory neuron excitability [23]. This suppression of excitability was *not* related to inhibition of sodium channels but rather linked to impairment of T-type (CaV3.x) channel function [23]. T-type VGCCs are low voltage-activated calcium channels important for the initiation of an action potential [1,24,25]. In neuropathic pain, CaV3.2 channels were reported to be increased at presynaptic sites through a trafficking mechanism [26]. Thus, the observations that (1) a peptide derived from CRMP2 could inhibit T-type VGCCs [23] and (2) T-type VGCCs are increased in a model where CRMP2 is dysregulated, together support the hypothesis that CRMP2 could modulate T-type VGCCs.

To directly test whether CRMP2 could regulate T-type calcium currents in DRG sensory neurons, we used a previously validated short interfering RNA (siRNA) against CRMP2 [19,27] to knockdown CRMP2 protein levels. Rat DRG neurons were co-transfected with CRMP2 siRNA or control siRNA and (~50 ng) of green fluorescent protein (GFP) expressing plasmid to identify the transfected cells prior to electrophysiological recordings. We used electrophysiology protocols described before [24] to record T-type currents. From a holding potential of −90 mV, we used 200-ms depolarization steps to change the membrane potential from −70 to +60 mV (10 mV increments) to evoke prototypical T-type calcium currents (Figure 1(a)). After transfection with CRMP2 (*n* = 15) or control (*n* = 17) siRNA, we recorded low voltage-activated calcium currents (Figure 1(a)) from DRG neurons with an average diameter between 20 and 30 µm. We measured current–voltage (*I*–*V*) relationships (Figure 1(b)) and observed that loss of CRMP2 expression had no effect on T-type calcium current amplitudes at all test potentials tested (Figure 1(b)). At peak current density (−10 mV), no difference was found between CRMP2 siRNA or control siRNA transfected DRG neurons (Figure 1(c) and Table 1). Knockdown of CRMP2 did not alter the channel gating properties as we measured a similar half-maximal activation (*V*_0.5_) of T-type calcium channels in both transfected conditions (Figure 1(d)). The kinetics of macroscopic current inactivation (Figure 1(e)) were unchanged at all membrane potentials tested (−40 mV, Figure 1(f)). The time-dependent activation (10–90% rise time) of T-type currents was not affected by loss of CRMP2 expression (Figure 1(g–h)). We next tested whether CRMP2 could control the voltage-dependent kinetics of channel inactivation (Figure 1(i)) and found this property to also not be affected by CRMP2 siRNA transfection. Deactivating tail currents calculated using the single exponential function: *y* = *A*_1_ × *e*(−*x*/*τ*_1_) + *y*_0_, where *A*_1_ is the amplitude, *τ*_1_ is the decay constant, and *y*_0_ is the offset. The resulting *τ* values (Figure 1(j)), showed no differences irrespective of CRMP2 expression. Finally, because upon long membrane hyperpolarizations in DRG neurons T-type calcium channels can recover from inactivation, we tested if this biophysical parameter could be contingent on CRMP2. This property has important consequences on the firing properties of sensory neurons expressing T-type calcium channels. Thus, we tested the recovery from inactivation using a double-pulse protocol with a variable interpulse duration at −90 mV (Figure 1(k)) after a 500-ms-long inactivating pulse (*V*_h_ = −90 mV; *V*_t_ = −30 mV). T-type currents recovered fully, independently of the transfection condition (Figure 1(k)). Taken together, our results show that CRMP2 has no role to play on regulation of T-type calcium currents and gating properties of these T-type calcium channels. We previously reported that CRMP2 has no effect on the R-type VGCC [13]. The present results showing the lack of CRMP2 regulation of T-type VGCC suggest now to support the assertion that CRMP2 is a specific regulatory protein for CaV2.2 but for no other type of VGCCs. This specificity is an important step in furthering our understanding of the contribution of CRMP2 in neuropathic pain.

**Table 1.** Gating properties of T-type calcium channels in DRG neurons.^a^

	Control	siRNA
Activation		
*V*_1/2_ (*P* = 0.10)	−22.7 ± 1.6(17)	−26.3 ± 1.4(16)
*k* (*P* = 0.42)	12.8 ± 1.7(17)	11.0 ± 1.4(16)
Inactivation		
*V*_1/2_ (*P* = 0.41)	−54.9 ± 1.1(18)	−53.6 ± 1.1(18)
*k* (*P* = 0.94)	−9.4 ± 1.0(18)	−9.5 ± 1.0(18)
Recovery		
*τ*_1_ (ms) (*P* = 0.87)	1036.0 ± 217.2(15)	981.0 ± 255.1(18)
*τ*_2_ (ms) (*P* = 0.82)	25.2 ± 4.8(15)	27.2 ± 6.9(18)

^a^Values are means ± SEM calculated from fits of the data from the indicated number of individual cells (in parentheses) to the Boltzmann equation; *V*_1/2_ midpoint potential (mV) for voltage-dependent activation or inactivation; *k*, slope factor. *τ*_1_ and *τ*_2_, time constants; the data could only be fit with a double exponential equation as reported by the Todorovic group [24] and *P* > 0.05; Student’s *t* test.

**Figure 1.**
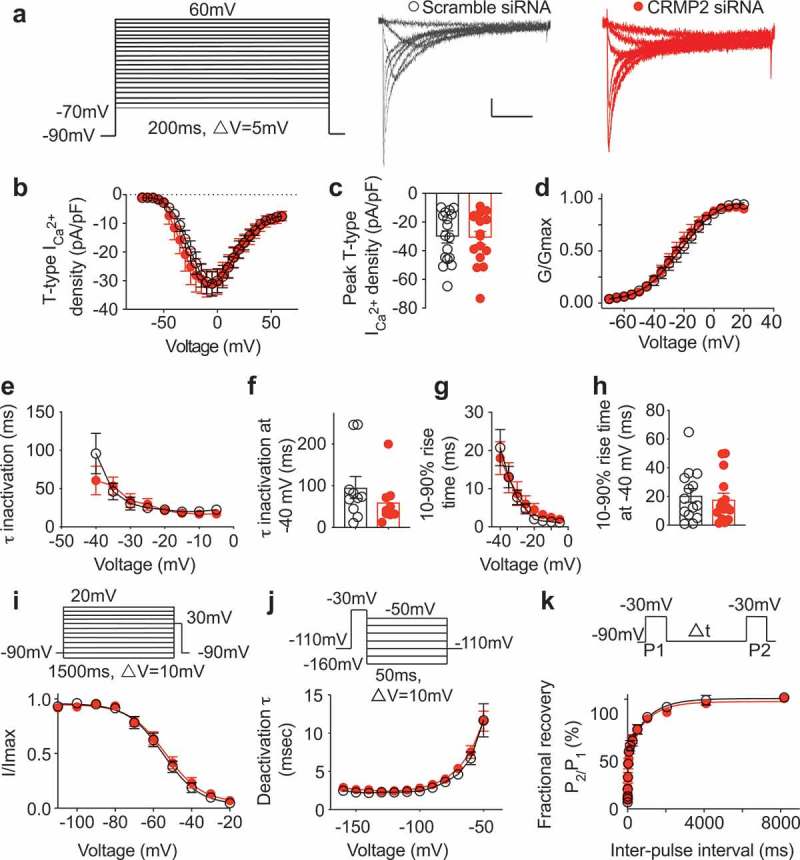
**CRMP2 does not affect T-type Ca**^2**+**^ **currents in dorsal root ganglion (DRG) sensory neurons**. (a) Representative family of traces of T-type Ca^2+^ currents from DRG sensory transfected with either control or CRMP2 siRNA. Voltage protocol used to evoke the currents is shown. Summary of the normalized (pA/pF) T-type calcium current density versus voltage relationship (b) and peak T-type Ca^2+^ current density at −10 mV (mean ± SEM) (c) from DRG sensory neurons transfected as indicated. (d) Boltzmann fits for normalized conductance *G*/*G*_max_ voltage relations for voltage-dependent activation of T-type currents. (e) Inactivation *τ* (single-exponential fit of decaying portion of the current waveforms using a single-exponential equation: *y* = *A*_1_ × *e*(−*x*/*τ*_1_) + *y*_0_, where *A*_1_ is the amplitude, *τ*_1_ is the decay constant, and *y*_0_ is the offset), isolated at −40 mV (f) and (g) time-dependent activation (10–90% rise time) from *I*–*V* curves and at −40 mV (h) in DRG cells shown in (b). Boltzmann fits for normalized conductance *G*/*G*_max_ voltage relations for voltage-dependent inactivation (i) of sensory neurons treated as indicated. (j) Deactivating tail currents in DRG neurons transfected with control or CRMP2 siRNA were fit with a single-exponential function. The resulting *τ* values are plotted. (k) Recovery from inactivation in indicated groups. Data are averaged and fitted by double exponential association (*P* > 0.05, *n* > 12 per condition). All graphs show mean ± SEM with individual data points showed when possible.

We conclude that CRMP2 regulation of NaV1.7 [9,10,12,13,22,28] is responsible for controlling DRG neurons excitability and that CRMP2 regulation of CaV2.2 is responsible for increased neurotransmitter release at the primary afferent [11,13,17,18]. For future therapeutic targeting, the specificity of action of CRMP2 toward CaV2.2 will reduce the potential for unwanted effects of novel CRMP2-targeted compounds.

## Methods

### Preparation of rat DRG cultures (acutely dissociated neurons)

DRG neurons were cultured using methods as described previously [29]. Collected dorsal root ganglia were digested in an enzymatic combination containing bicarbonate-free, serum-free, sterile DMEM (Cat# 11965, Thermo Fisher Scientific) solution, neutral protease (3.125 mg/mL, Cat#LS02104; Worthington, Lakewood, NJ), and collagenase type I (5 mg/mL, Cat# LS004194, Worthington, Lakewood, NJ). After incubation for approximately 1 h with gentle agitation under 37°C, DRG neurons were centrifuged post-dissociation and isolated from DRG media (DMEM containing 1% penicillin/streptomycin sulfate from 10,000 μg/mL stock, 30 ng/mL nerve growth factor, and 10% fetal bovine serum [Hyclone]). Dissociated DRG neurons were subsequently plated onto 12- mm laminin and poly-d-lysine-coated coverslips. Cultures were utilized before 2-days’ time.

### Transfection of rat primary DRG neurons

Collected cells were resuspended in nucleofector transfection reagent containing 6 μL (50 nM) scrambled or CRMP2 siRNA plus 6 µL GFP (0.5 μg/μL). Then, cells were subjected to electroporation protocol O-003 in an Amaxa Biosystems (Lonza) and plated onto 12- mm poly-d-lysine- and laminin-coated glass coverslips.

### Whole-cell electrophysiological recordings of calcium currents in acutely dissociated DRG neurons

The protocol for isolating T-type calcium currents was previously described by Choe et al. [24]. The extracellular recording solution used to isolate T currents consisted of the following (in millimolar): 2CaCl_2_, 152 TEA-Cl, 10 HEPES, pH adjusted to 7.4 with TEA-OH. The intracellular recording solution consisted of (in millimolar) 135 tetramethylammonium hydroxide, 10 EGTA, 40 HEPES, and 2 MgCl_2_, pH adjusted to 7.2 with hydrofluoric acid. As previously described, the majority of acutely dissociated small DRG cells express T currents [24]. Thus, in order to record T-type calcium currents, we focused on small DRG neurons with an average soma diameter of 20–30 μm.

To avoid contamination by residual High Voltage Activated (HVA) currents which are present at more positive membrane potentials, we measured the T-type Ca^2+^ current from the peak, which was subtracted from the current at the end of the depolarizing test potential. Activation of *I*_Ca-T_ was measured by using a holding voltage of −90 mV with voltage steps 200 ms in duration applied at 500-ms intervals in 10 mV increments from −70 to +60 mV. Inactivation of *I*_Ca-T_ was determined by applying an 1500-ms conditioning prepulse (−110 to +20 mV in 10 mV increments) after which the voltage was stepped to −30 mV for 20 ms; a 40-ms interval with a holding voltage of −90 mV separated each acquisition. In the deactivation tau protocol, the neuron was first hold at −110 mV, then the voltage jumped to −30 mV for 10 ms followed by a 50-ms conditioning prepulse (−160 to −40 mV in 10 mV increments). A 2-s interval with a holding voltage of −90 mV separated each acquisition. *I*_Ca-T_ recovery from inactivation was obtained by using our standard double-pulse protocol with variable interpulse duration at −90 mV after a 500-ms-long inactivating pulse (*V*_h_ = −90 mV; *V*_t_ = −30 mV).

The Boltzmann relation was used to determine the voltage dependence for activation of *I*_Ca_ wherein the conductance–voltage curve was fit by the equation *G*/*G*_max_ = 1/[1+ exp (*V*_0.5_ − *V*_m_)/*k*], where *G* is the conductance *G* = *I*/(*V*_m_ − *E*_Ca_), *G*_max_ is the maximal conductance obtained from the Boltzmann fit under control conditions, *V*_0.5_ is the voltage for half-maximal activation, *V*_m_ is the membrane potential, and *k* is a slope factor. *E*_Ca_ is the reversal potential for *I*_Ca_. The values of *I*_Ca_ around the reversal potential were fit with a linear regression line to establish the voltage at which the current was zero. The Boltzmann parameters were determined for each individual neuron and then used to calculate the mean ± SEM.
